# Experimental studies of paranoid thinking in clinical and nonclinical populations: a systematic review and meta-analysis

**DOI:** 10.1017/S0033291723001708

**Published:** 2023-10

**Authors:** Lyn Ellett, Filippo Varese, Jane Owens, Sonya Rafiq, Georgia Penn, Katherine Berry

**Affiliations:** 1 School of Psychology, University of Southampton, UK; 2 Division of Psychology and Mental Health, School of Health Sciences, Manchester Academic Health Science Centre, University of Manchester, UK; 3 Complex Trauma and Resilience Research Unit, Greater Manchester Mental Health NHS Foundation Trust, UK; 4 NHS Lothian, Edinburgh, UK

**Keywords:** Experiment, induction, meta-analysis, paranoia, review (systematic)

## Abstract

Paranoia is common in clinical and nonclinical populations, consistent with continuum models of psychosis. A number of experimental studies have been conducted that attempt to induce, manipulate or measure paranoid thinking in both clinical and nonclinical populations, which is important to understand causal mechanisms and advance psychological interventions. Our aim was to conduct a systematic review and meta-analysis of experimental studies (non-sleep, non-drug paradigms) on psychometrically assessed paranoia in clinical and nonclinical populations. The review was conducted using PRISMA guidelines. Six databases (PsycINFO, PubMed, EMBASE, Web of Science, Medline and AMED) were searched for peer-reviewed experimental studies using within and between-subject designs to investigate paranoia in clinical and nonclinical populations. Effect sizes for each study were calculated using Hedge's *g* and were integrated using a random effect meta-analysis model. Thirty studies were included in the review (total *n* = 3898), which used 13 experimental paradigms to induce paranoia; 10 studies set out to explicitly induce paranoia, and 20 studies induced a range of other states. Effect sizes for individual studies ranged from 0.03 to 1.55. Meta-analysis found a significant summary effect of 0.51 [95% confidence interval 0.37–0.66, *p* < 0.001], indicating a medium effect of experimental paradigms on paranoia. Paranoia can be induced and investigated using a wide range of experimental paradigms, which can inform decision-making about which paradigms to use in future studies, and is consistent with cognitive, continuum and evolutionary models of paranoia.

## Introduction

Paranoia, or persecutory ideation, occurs when a person believes they are under intentional threat of harm from others (Freeman & Garety, 2000). While paranoid delusions are often cited as the most commonly experienced form of delusional thinking within clinical populations, substantial evidence suggests that paranoid thinking commonly occurs in the general population (Garety, Everitt, & Hemsley, 1988; Ellett, Lopes, & Chadwick, 2003; Bebbington et al., 2013; Freeman et al., 2021). This is consistent with current dimensional models of mental ill-health (Caspi et al., 2013). A number of psychosocial models have been proposed that place varying emphasis on the importance of developmental, cognitive, behavioural, affective and interpersonal factors involved in the formation and maintenance of persecutory beliefs. Cognitive models emphasise the importance of the interpretation of anomalous events, and how these interpretations are influenced by factors such as previous life experience, attentional and attributional biases and affective states (Garety, Kuipers, Fowler, Freeman, & Bebbington, 2001; Morrison, 2001; Freeman, Garety, Kuipers, Fowler, & Bebbington, 2002; Bentall, Corcoran, Howard, Blackwood, & Kinderman, 2011).

Psychological research using methodologies for inducing and/or measuring paranoid thinking in both clinical and nonclinical samples offers a unique approach to the study of paranoia. These methodologies have been used to test theoretical models of paranoia by investigating factors that buffer or attenuate experiences of paranoia (Ellett & Chadwick, 2007; Lincoln, Hohenhaus, & Hartmann, 2013). However, they have been used most extensively in analogue samples, which provide a convenient and acceptable means of testing psychosocial models of paranoia given dimensional models of mental ill-health and paranoia in particular. Their use in clinical samples is relatively less well developed but an important area of research, most notably, in testing the role of potential buffers and protective factors in relation to paranoid thinking. Despite the popularity of paranoia induction methodologies, there is currently no meta-analysis of experimental studies of paranoia which can guide researchers in designing and evaluating research in this area. Two recent systematic reviews, one investigating the role of anxiety in paranoia and one investigating a range of causal mechanisms in delusions and hallucinations, included a review of studies using experimental procedures to manipulate paranoia (Bennetts, Stopa, & Newman-Taylor, 2021; Brown, Waite, & Freeman, 2019). However, these reviews did not meta-analyse the effects of the experimental procedures on outcomes, which is important to establish the size of the effects. The aim of the current review was to summarise the existing literature on experimental paradigms that have been used to study paranoid thinking in both clinical and nonclinical populations. Given the proposed structure of paranoia as potentially including both persecutory ideation and/or social reference (Bebbington et al., 2013; Bell & O'Driscoll, 2018), we chose to focus on the facet of paranoid thinking specifically. Additionally, given our main focus was to summarise the literature on experimental studies that measured paranoia as an outcome, we did not use a specific theoretical model to guide our decision-making about which paradigms to include, as this could have potentially limited the scope of studies included in the review. The current systematic review and meta-analysis will, therefore, address the following central questions: (1) What experimental paradigms have been used to induce or study paranoia?; (2) How effective are these experimental paradigms?; (3) What factors mediate and moderate the effect of experimental paradigms on paranoia?

## Method

Six databases (PsycINFO, PubMed, EMBASE, Web of Science, Medline and AMED) were searched up to December 2021. Abstracts and titles were searched for the following: (persecution OR persecutory OR paranoid OR paranoia OR suspiciousness OR suspicious thoughts) AND (experimental OR manipulation OR manipulated OR induction OR induced OR paradigm).

Inclusion criteria for the review were as follows: (1) studies that used an experimental design to induce, manipulate or measure paranoid thinking; (2) clinical or nonclinical population and (3) studies that assessed paranoia using a psychometric measure. Exclusion criteria for the review were as follows: (1) studies that measured factors conceptually related to but different from paranoia, such as reasoning biases, attributions of harmful intent or other types of delusions or hallucinations, because the focus of the review was on the assessment of paranoia using psychometric measures; (2) studies where the sample included participants under 18 years old; (3) studies assessing suspiciousness of experimental procedures; (4) experimental studies of drug or sleep induced paranoia, or the effect of hearing deficits because the focus here was on psychological approaches; (5) studies that did not include a reliable and valid measure of paranoia, such as unstandardized visual analogue scales, interview-based ratings or single scale items; (6) studies that used correlational or cross-over designs due to the risk of carryover effects; (7) studies that did not provide sufficient data to enable us to calculate effects sizes (in the case of within subjects designs pre- and post-induction scores on a measure of paranoia and in the case of between subjects’ designs post-induction scores on measure of paranoia for an experimental and control group within or without adjustments for baseline scores) (Martin, 1970; Cook & Perrin, 1971; Locascio & Snyder, 1975; Zimbardo, Andersen, & Kabat, 1981; Horvat, 1986; Casanova, Katkovsky, & Hershberger, 1988; Green, Nuechterlein, & Breitmeyer, 1995; Couzoulis-Mayfrank et al., 2005; Kahn-Greene, Killgore, Kamimori, Balkin, & Killgore, 2007; Mason, Morgan, Stefanovic, & Curran, 2008). Where data were not reported in the papers, we attempted to obtain this from the authors.

**Figure 1.**
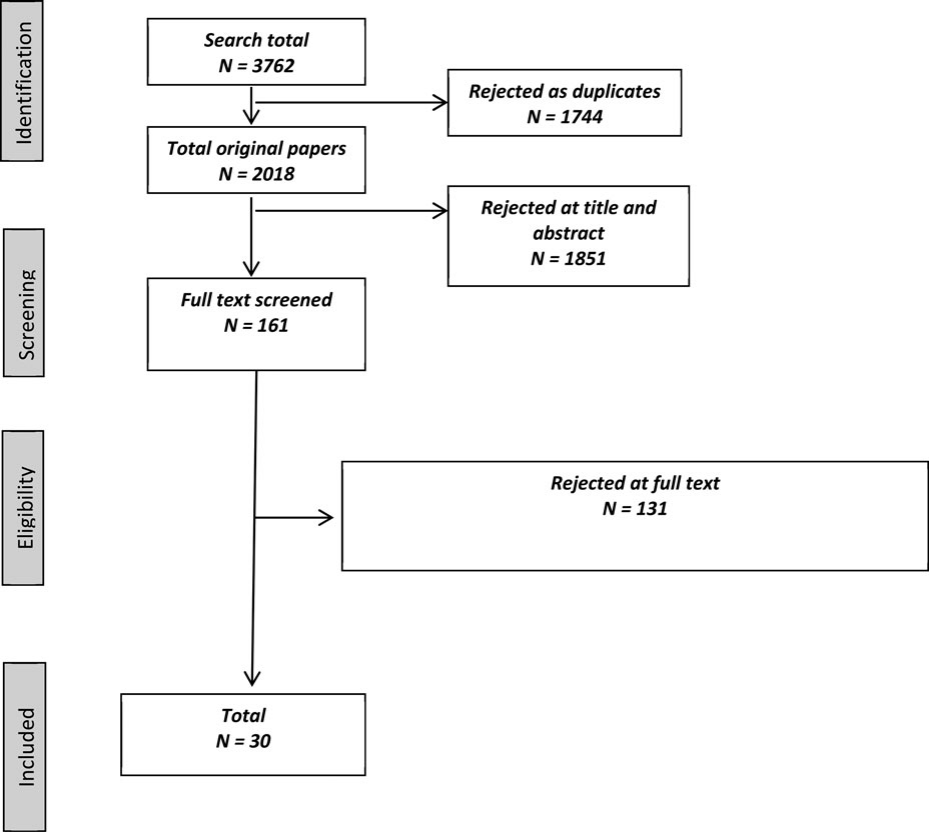
Search results (Figure adapted from guidelines set out by the PRISMA group).

Of the studies that fulfilled our inclusion criteria, only English language and peer-reviewed articles were included, and no restriction was placed on the year of publication. Following the exclusion of duplicate articles, each paper was assessed at either title, abstract or full text level to determine suitability. Some studies were excluded from the review for multiple reasons, making it difficult to allocate each study to a single reason for exclusion. However, the most common reasons for exclusion were not including a post-manipulation or validated measure of paranoia, not employing an experimental design, use of a biological paradigm (e.g. sleep and drug studies), irrelevant topic, not published in the English language and case studies. The review included 30 papers, and Fig. 1 summarises the search process.

### Effect size data extraction and statistical analyses

CMA Version 3 was used for all statistical analyses. All effect size computations were conducted using CMA except for the within-subject effects for which no pre–post correlation was available. For studies employing a between-subject/independent group design, effect sizes of the d-family were calculated using standard computational approaches from reported post-manipulation means, standard deviations and sample sizes (Borenstein, Hedges, Higgins, & Rothstein, 2009). For studies employing a within-subject/paired group design, effect sizes were calculated based on pre- and post-manipulation means, standard deviations and sample size as well as the reported correlation coefficient between pre- and post-manipulation scores (Borenstein et al., 2009). When the correlation coefficient was not reported, the review team requested the missing information by contacting the study authors. To maximise the number of effects included in the planned analyses, when study authors were unable to provide the missing correlation coefficient, we employed the effect size calculation approach (Hirst, Cragg, & Allen, 2018). When studies reported the effect of paranoia induction manipulations in multiple, independent groups, effect sizes were calculated for each independent sample and included in the analyses as separate effects. In all cases, the Hedges' correction was applied to the computed effects ahead of statistical integration via a random-effect meta-analysis.

Effect sizes (i.e. Hedges' *g*) were integrated using the random effect meta-analysis. Due to the considerable heterogeneity in the design of the included studies, prior to the main analysis, we conducted a subgroup analysis to explore potential systematic differences in effect sizes of studies employing a within-subject/paired group design and those employing a between-subject/independent group design. All available effects were then considered into a single analysis to investigate the overall effect of paranoia induction procedures in the available literature. This principal analysis was followed-up through subgroup analyses to explore and summarise descriptive differences in the summary effects obtained by studies employing different paranoia induction procedures. Statistical heterogeneity was examined and quantified using the *Q* test and *I*^2^ statistic, and influence analyses (one-study-removed analyses) were conducted to identify single studies associated with summary effects. Publication bias was assessed for through visual examination of funnel plots and the results of Egger's test. Egger's tests were conducted using the significance level of 0.10 instead of the conventional 0.05 due to limited studies in the meta-analysis resulting in limited power of statistical tests for funnel plot asymmetry (Egger, Smith, Schneider, & Minder, 1997). When appropriate, the Duval and Tweedie ‘Trim and Fill’ method was used to adjust the results for the potential impact of hypothetically missing studies (Duval & Tweedie, 2000).

## Results

### Summary of studies

Thirty studies met criteria for inclusion in the review (total *n* = 3898). Preliminary data extracted from studies are presented in Table 1. Across studies, a range of paranoia measures (*n* = 4), induced states (*n* = 12) and methodological paradigms (*n* = 13) were used. Study designs included both between (*n* = 14) and within-subjects (*n* = 16), and studies employed a nonclinical sample (*n* = 26), a clinical and nonclinical sample (*n* = 3) or a clinical sample only (*n* = 1). Clinical samples included patients with psychotic disorders, persecutory delusions, anxiety disorders or people at risk of developing psychosis.

**Table 1.** Study Characteristics and Effect Sizes

Study	Measure	Induced state	Methodology	Hedge's *g*	Variance	LL	UL
Bennetts et al. (2020)	Paranoia checklist	Suspicion/mistrust	Imagery	0.660	0.047	0.233	1.087
Boden and Berenbaum (2007)	Paranoia scale	Emotional awareness	Stories	−0.417	0.035	−0.782	−0.053
Bodner and Mikulincer (1998)	Bodner state paranoia	Paranoia	Attentional focus	0.624	0.210	−0.273	1.522
Bullock et al. (2016)	Paranoia checklist	Suspicion/mistrust	Imagery	1.894	0.134	1.176	2.612
Butler et al. (2019) sample 1	Bodner state paranoia	Social exclusion	Cyberball	0.393	0.029	0.059	0.726
Butler et al. (2019) sample 2	Bodner state paranoia	Social exclusion	Cyberball	0.030	0.029	−0.302	0.362
Ellett and Chadwick (2007) sample 1	Paranoia scale	Paranoia	Attentional focus	1.101	0.230	0.160	2.041
Ellett and Chadwick (2007) sample 2	Bodner state paranoia	Paranoia	Attentional focus	1.091	0.115	0.427	1.755
Ellett et al. (2008)	State social paranoia scale	Paranoia	Exposure to urban environment	1.552	0.173	0.736	2.368
Flower et al. (2015)	Bodner state paranoia	Paranoia	Attentional focus	1.595	0.094	0.994	2.196
Gollwitzer et al. (2018)	Paranoia checklist	Loneliness	Recall of specific loneliness situation	0.289	0.027	−0.035	0.613
Hepper et al. (2021)	Bodner state paranoia	Social exclusion	Cyberball	0.588	0.009	0.404	0.772
Hutton et al. (2017)	Bodner state paranoia	Paranoia	Attentional focus	0.600	0.010	0.408	0.792
Kaltsi et al. (2018)	Paranoia checklist	Social exclusion	Cyberball	1.400	0.002	1.319	1.481
Kesting et al. (2013)	Paranoia checklist	Social exclusion	Cyberball	0.282	0.053	−0.171	0.734
Kingston and Ellett (2014)	Bodner state paranoia	Paranoia	Attentional focus	0.354	0.024	0.053	0.655
Lamster et al. (2017)	Paranoia checklist	Loneliness	Manipulated questionnaire + feedback	0.685	0.118	0.012	1.357
Lincoln et al. (2010)	Paranoia checklist	Anxiety	Exposure to anxiety-inducing pictures	0.661	0.047	0.236	1.085
Lincoln et al. (2018) sample 1	Paranoia checklist	Social exclusion	Cyberball	0.330	0.002	0.236	0.424
Lincoln et al. (2018) sample 2	Paranoia checklist	Social exclusion	Cyberball	0.540	0.002	0.443	0.637
Lincoln et al. (2018) sample 3	Paranoia checklist	Social exclusion	Cyberball	0.660	0.007	0.499	0.821
Lopes and Jaspal (2015) sample 1	State social paranoia scale	Social stress	Video clips	0.202	0.077	−0.343	0.746
Lopes and Jaspal (2015) sample 2	State social paranoia scale	Social stress	Video clips	−0.075	0.060	−0.555	0.406
Lopes and Pinto-Gouveia (2012) sample 1	State social paranoia scale	Failure	Computer game	−1.061	0.064	−1.556	−0.567
Lopes and Pinto-Gouveia (2012) sample 2	State social paranoia scale	Failure	Computer game	0.871	0.045	0.458	1.285
Lopes and Pinto-Gouveia (2012) sample 3	State social paranoia scale	Failure	Computer game	1.162	0.059	0.687	1.637
Martinelli et al. (2013)	State social paranoia scale	Rumination	Cognitive coping strategy task	0.200	0.010	0.009	0.391
Marr et al. (2011)	Work-adapted paranoia scale	Relationship threat	Goal induction	0.604	0.067	0.095	1.113
Newman-Taylor et al. (2021)	Paranoia checklist	Insecure attachment	Imagery	−0.037	0.048	−0.468	0.394
Prévost et al. (2011) sample 1	Bodner state paranoia	Paranoia	Attentional focus (mirror)	0.490	0.014	0.260	0.720
Prévost et al. (2011) sample 2	Bodner state paranoia	Paranoia	Attentional focus (mirror)	1.010	0.013	0.782	1.238
Sellers et al. (2018) sample 1	Paranoia checklist	Metacognitive beliefs about uncontrollability and dangerousness of thoughts	Video clips	0.360	0.003	0.258	0.462
Sellers et al. (2018) sample 2	Paranoia checklist	Metacognitive beliefs about uncontrollability and dangerousness of thoughts	Video clips	0.260	0.003	0.158	0.362
Soflau and David (2019)	State social paranoia scale	Irrational beliefs	Virtual reality	1.392	0.061	0.907	1.878
Sood and Newman-Taylor (2020)	Paranoia checklist	Paranoia/threat	Imagery	0.110	0.001	0.042	0.178
Sood et al. (2021) sample 1	Paranoia checklist	Secure/anxious/avoidant attachment	Imagery	0.190	0.000	0.150	0.230
Sood et al. (2021) sample 2	Paranoia checklist	Secure/anxious/avoidant attachment	Imagery	0.280	0.000	0.240	0.320
Sood et al. (2021) sample 3	Paranoia checklist	Secure/anxious/avoidant attachment	Imagery	0.485	0.000	0.447	0.523
Sundag et al. (2018) sample 1	Paranoia checklist	Social exclusion	Cyberball	0.670	0.010	0.478	0.862
Sundag et al. (2018) sample 2	Paranoia checklist	Social exclusion	Cyberball	0.540	0.002	0.443	0.637
Veling et al. (2016)	State social paranoia scale	Social stress	Virtual reality	0.970	0.000	0.950	0.990
Westermann et al. (2012)	Paranoia checklist	Social exclusion	Cyberball	0.121	0.035	−0.243	0.486

### Methodological paradigms

Two of the authors (L.E. and K.B.) independently grouped the studies based on methodological paradigm employed, resulting in 100% agreement for the following groupings: *Cyberball* (*k* = 7; Westermann, Kesting, & Lincoln, 2012; Kesting, Bredenpohl, Klenke, Westermann, & Lincoln, 2013; Kaltsi, Bucci, & Morrison, 2018; Lincoln, Sundag, Schlier, & Karow, 2018; Sundag, Ascone, & Lincoln, 2018; Butler, Berry, Ellett, & Bucci, 2019; Hepper, Wildschut, Sedikides, Robertson, & Routledge, 2021), *imagery* (*k* = 5; Bullock, Newman-Taylor, & Stopa, 2016; Bennetts, Stopa, & Newman-Taylor, 2020; Sood & Newman-Taylor, 2020; Sood, Carnelley, & Newman-Taylor, 2021; Newman-Taylor, Sood, Rowe, & Carnelley, 2021), *attentional focus* (*k* = 6; Bodner & Mikulincer, 1998; Ellett & Chadwick, 2007; Prévost et al. 2011; Kingston & Ellett, 2014; Flower, Newman-Taylor, & Stopa, 2015; Hutton, Ellett, & Berry, 2017), *virtual reality* (*k* = 2; Veling, Counotte, Pot-Kolder, van Os, & van der Gaag, 2016; Soflau & David, 2019), *videos* (*k* = 2; Lopes & Jaspal, 2015; Sellers, Wells, & Morrison, 2018), *situational recall of a loneliness experience* (*k* = 1; Gollwitzer et al., 2018); *manipulated questionnaire with feedback* (*k* = 1; Lamster, Nittel, Rief, Mehl, & Lincoln, 2017); *exposure to anxiety pictures* (*k* = 1; Lincoln, Lange, Burau, Exner, & Moritz, 2010), *stories* (*k* = 1; Boden & Berenbaum, 2007), *cognitive coping strategy task* (*k* = 1; Martinelli, Cavanagh, & Dudley, 2013); *exposure to urban environment* (*k* = 1; Ellett, Freeman, & Garety, 2008), *goal induction* (*k* = 1 Marr, Thau, Aquino, & Barclay, 2012) and *computer game* (*k* = 1; Lopes & Pinto-Gouveia, 2012).

### Induced states

In total, 10 studies set out to explicitly induce paranoia, and 20 studies induced a range of other states, including social exclusion (*k* = 7); loneliness (*k* = 2); social stress (*k* = 2); attachment (*k* = 2); anxiety (*k* = 1); emotional awareness (*k* = 1); failure (*k* = 1); relationship threat (*k* = 1); metacognitive beliefs (*k* = 1); irrational beliefs (*k* = 1) and rumination (*k* = 1).

### Paranoia measures

A range of measures were used across the studies to measure paranoia including original or state version of the Paranoia Checklist (Freeman et al., 2005) (*k* = 14), state paranoia as originally reported in Bodner and Mikluciner (1998) (*k* = 8), state social paranoia scale (Freeman et al., 2007) (*n* = 6), Paranoia Scale (Fenigstein & Vanable, 1992) (*n* = 2) and a work-adapted version of the Paranoia Scale (Marr et al., 2012) (*n* = 1) (note *n* for paranoia measures is 31 as one study used two different measures).

### Quality appraisal of included studies

We used three criteria that we deemed important to the integrity of experimental paradigms to assess the quality of the studies included in the review: design, sensitivity of measures to change and representativeness of the sample. We then rated each study high, medium or low risk of bias in terms of each of these three criteria Design (high risk = groups not randomised; medium risk = groups randomised; low risk = within subjects design); Sensitivity to change, based on whether a state as opposed to a trait paranoia measure was used (high risk = trait measure used, low risk = state measure used) and representativeness of the sample (high risk = student population, medium risk = general population or clinical, not randomly selected, low risk = general population or clinical, randomly selected). See Table 2 for a breakdown of scoring.

**Table 2.** Quality Appraisal of Studies

Study	Design	Sensitivity to change	Sample representativeness
Bennetts et al. (2021)	Low risk	Low risk	High risk
Boden and Berenbaum (2007)	Medium risk	High risk	High risk
Bodner and Mikulincer (1998)	Medium risk	Low risk	High risk
Bullock et al. (2016)	High risk	Low risk	High risk
Butler et al. (2019)	Low risk	Low risk	High risk
Ellett and Chadwick (2007)	Medium risk	High risk	High risk
Ellett et al. (2008)	Medium risk	Low risk	Medium risk
Flower et al. (2015)	High risk	Low risk	High risk
Gollwitzer et al. (2018)	Medium risk	Low risk	Medium risk
Hepper et al. (2021)	Medium risk	Low risk	Medium risk
Hutton et al. (2017)	Low risk	Low risk	High risk
Kaltsi et al. (2018)	Low risk	Low risk	High risk
Kesting et al. (2013)	Medium risk	Low risk	Medium risk
Kingston and Ellett (2014)	Low risk	Low risk	High risk
Lamster et al. (2017)	Medium risk	Low risk	Medium risk
Lincoln et al. (2010)	Medium risk	Low risk	High risk
Lincoln et al. (2018)	Low risk	Low risk	Medium risk
Lopes and Jaspal (2015)	Medium risk	Low risk	High risk
Lopes and Pinto-Gouveia (2012)	Low risk	Low risk	High risk
Martinelli et al. (2013)	Low risk	Low risk	High risk
Marr et al. (2012)	Medium risk	High risk	Medium risk
Newman-Taylor et al. (2021)	Medium risk	Low risk	Medium risk
Prévost et al. (2011)	Low risk	Low risk	Medium risk
Sellers et al. (2018)	Low risk	Low risk	High risk
Soflau and David (2019)	Medium risk	Low risk	High risk
Sood and Newman-Taylor (2020)	Low risk	Low risk	Medium risk
Sood et al. (2021)	Low risk	Low risk	Medium risk
Sundag et al. (2018)	Medium risk	Low risk	Medium risk
Veling et al. (2016)	Low risk	Low risk	Medium risk
Westermann et al. (2012)	Low risk	Low risk	Medium risk

Overall, the quality of studies included in the review was good, with the majority of the studies using either a randomised or within subjects design and nearly all studies using a state measure of paranoia. Studies were, however, weaker in terms of the representativeness of the sample using either student samples or non-randomly selected general population or clinical samples.

### Meta-analysis findings

**Figure 2.**
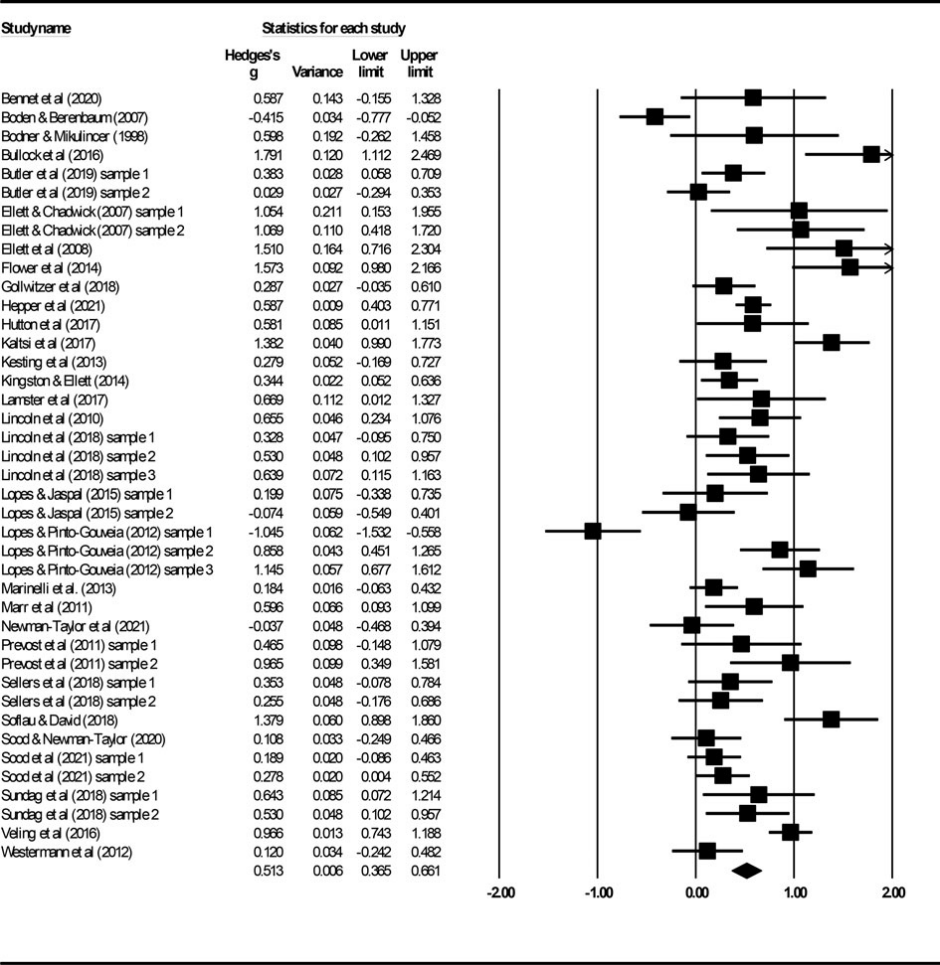
Forrest plot of the main analysis considering the overall effect of paranoia induction.

The subgroup analysis conducted ahead of planned primary analyses to investigate potential differences in effect sizes for studies with different designs indicated that the effects observed in between-subject/independent group studies [*k* = 16; Hedges' *g* = 0.58, 95% confidence interval (CI) 0.32–0.83] were not significantly different from those estimated for within-subject/paired group studies (*k* = 25; Hedges' *g* = 0.48, 95% CI 0.29–0.67; *Q* test = 0.39, *p* = 0.54). The analysis to evaluate the overall effect of paranoia induction procedures included effects extracted from 41 separate samples; the results are displayed in Fig. 2. The analysis found a significant summary effect of 0.51 [95% CI 0.37–0.66, *p* < 0.001]. Based on Cohen's (1988) criteria, this indicates that experimental paradigms have, on average, a ‘medium’ effect on paranoia. However, the results of heterogeneity analyses highlighted high levels of statistical heterogeneity; *Q*(40) = 212.60, *p* < 0.001; *I*^2^ = 81.19%. Despite the inclusion of studies with an uncharacteristic, statistically significant negative effect, one-study-removed analyses did not identify any study exerting undue influence on the estimated summary effect (Boden & Berenbaum, 2007; Lopes & Pinto-Gouveia, 2012). Inspection of funnel plots and the results of Egger's test (*p* = 0.26) did not suggest that the findings were affected by possible publication or other selection bias, and the application of the Trim and Fill method was, therefore, deemed unnecessary.

The results of subgroup analyses that explored separately the summary effects of different types of methodological paradigm are reported in Table 3.

**Table 3.** Subgroup Analyses Considering Specific Paranoia Induction Manipulations

Methodology or induced state	Point estimate (*g*)	LL	UL	*p*	*Q* test^a^	*I*^2*^
Anxiety induction	0.65	0.23	1.08	0.002	–	–
Attentional focus	0.84	0.41	1.27	<0.001	15.94, *p* = 0.007	68.63%
Cognitive coping strategy task	0.47	0.01	0.94	0.046	–	–
Computer game	0.32	−0.96	1.61	0.623	–	–
Cyberball	0.49	0.28	0.70	<0.001	35.30, *p* < 0.001	71.66%
Exposure to urban environment	1.51	0.72	2.30	<0.001	–	–
Goal induction	0.60	0.09	1.10	0.020	–	–
Imagery	0.40	0.04	0.75	0.027	23.07, *p* < 0.001	78.33%
Loneliness	0.37	0.06	0.67	0.018	–	–
Stories	−0.41	−0.78	−0.05	0.025	–	–
Video clips	0.19	−0.04	0.43	0.100	–	–
Virtual reality	1.12	0.73	1.50	<0.001	–	–

*Note.* Heterogeneity analysis is reported only for analyses with *k* > 5 due to likely bias in the estimation of statistical heterogeneity in meta-analyses with limited numbers of included studies.

### Mediators and moderators

A summary of mediation and moderation effects reported across studies is shown in Table 4. A number of mediators and moderators of the effect of the experimental paradigms on paranoia were tested in five studies and included negative affect, self-esteem, jumping to conclusions reasoning bias, cognitive fusion and beliefs about self and others (Lincoln et al., 2010; Kesting et al., 2013; Gollwitzer, Wilczynska, & Jaya, 2018; Sood & Newman-Taylor, 2020; Sood et al., 2021). Studies examining causal mechanisms found evidence that the effect of social exclusion on paranoia is mediated by self-esteem (small effect; Kesting et al., 2013); the impact of imagery on paranoia is mediated by cognitive fusion (large effect) and negative self and other beliefs (medium effect; Sood & Newman-Taylor, 2020; Sood et al., 2021); the association between anxiety and paranoia is mediated by an increase in the tendency to jump to conclusions (medium effect; Lincoln et al., 2010), and the effect of loneliness on paranoia is mediated by the negative affect (medium effect; Gollwitzer et al., 2018).

**Table 4.** Summary of Mediation and Moderation Effects

Mediation effects			
Study	Direct effect of condition on paranoia (*β*)	Mediators	Indirect effect (*β*)
Gollwitzer et al (2018)	Loneliness (0.27)	Negative affect	0.19
Kesting et al (2013)	Social exclusion (0.32)	Self-esteem	0.21
Lincoln et al (2010)	Anxiety (0.32)	Jumping to conclusions	0.24
Sood and Newman-Taylor et al (2020)	Attachment	Cognitive fusion	0.17
Sood et al (2021)	Anxious *v.* secure attachment (0.62)	Cognitive fusion Negative self Negative other	0.40 0.16 0.17
Sood et al (2021)	Avoidant *v.* secure attachment (2.19)	Cognitive fusion Negative self Negative other	0.39 0.13 0.14
Moderation effects	Interaction term	Moderator	(*β*)
Kesting et al (2013)	Social exclusion × psychosis proneness	Psychosis proneness	−0.01
Lamster et al (2017)	Change in loneliness × psychosis proneness	Psychosis proneness	0.089
Lincoln et al (2010)	Anxiety × psychosis proneness	Psychosis proneness	0.22

*Note.* This table summarises two of the four conditions needed for a mediation effect to occur—when the IV has a significant effect on the DV in the absence of the mediator (direct effect of condition on paranoia column) and when the effect of the IV on the DV reduces when the mediator is added to the model (indirect effect column).

Psychosis proneness was investigated as moderators in three studies (Lincoln et al., 2010; Kesting et al., 2013; Lamster et al., 2017). Findings indicated that psychosis proneness moderated the effect of anxiety and loneliness paradigms on paranoia, such that anxiety resulted in higher paranoia among individuals with high existing vulnerability (Lincoln et al., 2010). Additionally, individuals with high psychosis proneness showed a larger reduction in paranoia resulting from a decrease in loneliness (Lamster et al., 2017). One study found that psychosis proneness did not moderate the effect of social exclusion on paranoia (Kesting et al., 2013).

## Discussion

In this paper, we conducted a systematic review and meta-analysis of the literature on experimental studies of paranoia, measured using established psychometric scales, with the aim of identifying experimental paradigms (non-drug and non-sleep paradigms), evaluating their effectiveness and identifying mediators and moderators of paranoia. Thirteen experimental paradigms were employed, the most common being cyberball, attentional focus and imagery, and 12 states were induced, the most common being paranoia and social exclusion. A range of paranoia measures were also used across studies, the most common being a state-adapted version of the Paranoia Checklist.

A range of antecedents of paranoia were also identified in terms of the states that were intended to be induced by the paradigms. While a third of studies (*n* = 10) had the explicit aim of inducing paranoia, the remaining studies (*n* = 20) induced other states that may lead to paranoia. These included a range of beliefs (metacognitive and irrational), emotions (anxiety, social stress, loneliness and emotional awareness), situational factors (social exclusion, attachment, failure and relationship threat) and cognitive processes (rumination) consistent with both the ABC model used in cognitive behavioural therapy and current cognitive models of psychosis (Garety et al., 2001; Freeman et al., 2002).

Findings from the meta-analysis indicated a medium effect size for the effect of experimental paradigms on paranoia across the 30 studies. As we might expect, results from the subgroup analyses tentatively suggest that experimental studies using clinical populations (exposure to urban environment and virtual reality) had the largest effect sizes. Additionally, in studies that used nonclinical populations only, attentional focus and anxiety induction paradigms had the largest cumulative effect sizes, although the subgroup analyses need to be interpreted cautiously given the small number of studies in some experimental groupings. Overall, the evidence from the studies reviewed suggests that paranoia can be induced using a range of different methodologies, which adds weight to the conceptualisation of paranoia being both a common and evolutionarily adaptive response to perceived threat (Ellett et al., 2003) and is consistent with continuum models of psychosis (Strauss, 1969; van Os, Hanssen, Bijl, & Ravelli, 2000).

A number of factors were shown to mediate or moderate the effects of experimental paradigms on paranoid thinking. Consistent with current models of paranoid thinking (Garety et al., 1988; Morrison, 2001; Freeman et al., 2002; Bentall et al., 2011; Freeman et al., 2021), identified mediators included negative affect, self-esteem, jumping to conclusions reasoning bias, cognitive fusion and beliefs about self and others and moderators included psychosis proneness. Consistent with the stress-vulnerability model (Zubin & Spring, 1977), existing psychosis proneness was shown to exacerbate the effect of experimental paradigms on state paranoia in two studies. Interestingly, and again consistent with the stress-vulnerability model, the evidence also suggests that paranoia can also be induced in those with low trait disposition when environmental threat is high.

There are a number of limitations that warrant consideration, with both the individual studies included and the review overall. In terms of individual studies, effect size calculations were not possible for studies that did not report adequate data to allow this or for uncontrolled or correlational designs, in which the analyses presented did not pertain specifically to the effectiveness of induction(s). We attempted to contact authors for missing data but not all authors responded meaning that some potentially relevant studies could not be included. The issue of power of individual studies was addressed through the use of meta-analysis; however, it was noteworthy that many of the studies had small sample sizes. Additionally, we were not able to take into account the psychometric properties of the paranoia measures used across studies in our analyses, which means that the review is silent about which measures of paranoia are more or less sensitive to change. Findings from the meta-analysis indicated high levels of statistical heterogeneity, which need to be considered when interpreting the findings from this review. The review only included papers in the English language, such that there may be other studies that were not considered, and we were not able to determine whether the effectiveness of paranoia inductions differed between diagnostic groups. Furthermore, the focus of the review was solely on experimental studies that induce or manipulate paranoid thinking; therefore, some important areas were not included, such as factors associated with paranoia, and the findings do not generalise to biological inductions of paranoia. The review may also be subject to publication bias as the gray literature was not searched.

### Recommendations for future research and clinical implications

The review highlights a number of areas for consideration in future research. There are some key methodological points that should be taken forward in future research, in particular, the inclusion of both baseline and post-manipulation measures of paranoia, and the measurement of affective states and cognitive processes that may also be activated via different methodological approaches. Although this review has highlighted that studies have started to examine the effect of affective states (e.g. anxiety) and cognitive processes (e.g. rumination) on paranoia, future studies might also determine the extent to which increases in paranoia occur in the presence *v.* absence of other affective states, and the extent to which increases in paranoia are accompanied by activation of theoretically relevant cognitive processes, such as worry. This research is important to identify the causal mechanisms that are implicated in paranoia. Additionally, although the review highlighted psychosis proneness as a moderator, the identification of additional moderators is needed in future research. The recent publication of the revised Green Paranoid Thoughts Scale (R-GPTS, Freeman et al., 2021) with severity cut offs will also allow more precision in sample selection and will facilitate the identification of nonclinical groups with elevated levels of trait paranoia for inclusion in future experimental studies. This is perhaps particularly important given the findings from the current review that individuals with existing vulnerability were more prone to the effects of experimental paradigms.

To date, the majority of experimental studies have relied almost exclusively on self-report measures, which may be subject to demand characteristics. Additional behavioural indices of paranoia have started to be identified and used in research studies (e.g. trust and distrust behaviours, behavioural or social avoidance and inequity aversion in economic contexts), and future research would be strengthened by the use of both self-report measures and behavioural indices of paranoia. In terms of the measures, it is also important to consider how sensitive paranoia scales are to change, particularly in non-clinical samples where data may be skewed. If scales are highly sensitive to change, it may be more difficult to determine whether a statistically significant change in a score is clinically or theoretically meaningful. Furthermore, although the paranoia scales have reasonable convergent validity with each other (possibly due to the fact that some of the scales were derived from each other), the issue of discriminant validity, construct validity and test re-test reliability is less well-researched.

Research has started to focus on identifying and experimentally testing factors that may buffer or attenuate paranoid thinking. To date, positive self-cognitions, self-affirmation, self-compassion, priming positive attachment and cognitive reappraisal have been shown to be effective (Ellett & Chadwick, 2007; Kingston & Ellett, 2014; Lincoln, Stahnke, & Moritz, 2014; Bullock et al., 2016; Gollwitzer et al., 2018). A related issue yet to be investigated is the stability of induced paranoia following manipulation. Although one study has shown that, once activated, paranoia is slow to dissipate, it would be interesting to determine the extent to which increases in paranoia reduce naturalistically, as has been shown cross-sectionally in relation to individual paranoid experiences or whether it is amenable to specific interventions, such as mindfulness, which have been shown to be helpful in clinical and nonclinical populations (Ellett & Chadwick, 2007; Chadwick, Hughes, Russell, Russell, & Dagnan, 2009; Ellett, 2013; Chadwick, Newman-Taylor, & Abba, 2005; Allen-Crooks & Ellett, 2014; Shore, Strauss, Cavanagh, Hayward, & Ellett, 2018; Ellett et al., 2020). Future research might also usefully identify participants who are immune to paranoia induction in the context of threat, so that resiliency factors can be explored and fostered or enhanced, in individuals who experience paranoia across the continuum, consistent with a recent focus on strength-based approaches in psychological interventions (Chadwick, 2006; Padesky & Mooney, 2012).

While there is a growing literature on paranoia induction in nonclinical populations, there have been disproportionately fewer experimental studies conducted with clinical populations, which is a knowledge gap in the literature that should be addressed in future research. Additionally, a key limitation of experimental research is that it can lack ecological validity. Although research has started to examine the extent to which more naturalistic environments or interpersonal events give rise to paranoia in nonclinical populations (e.g. Hepper, Ellett, Kerley, & Kingston, 2022; Ellett, Foxall, Wildschut, & Chadwick, 2023), more research is needed in clinical populations, including identification of the specific characteristics of the environment that either intensify or attenuate paranoia. Finally, while some of the antecedents of paranoia have been investigated, there have been relatively few studies that have examined the behavioural consequences of paranoia.

There are also a number of important clinical implications from the reviewed studies that warrant consideration. For example, the ease with which paranoia can be induced in nonclinical samples may provide a normalising framework for people experiencing persecutory delusions and could be used to highlight factors involved in the development and maintenance of paranoia to individuals with these distressing experiences. Interestingly, some of the methodologies employed have started to be used and tested directly as the basis for psychological interventions, in particular, virtual reality (Freeman et al., 2016; Freeman et al., 2019). Finally, studies in this area have begun to identify factors that attenuate or buffer paranoia, which could be tested and potentially translated for use with clinical populations.

## Conclusion

The findings of this review demonstrate that paranoia can be induced using a wide range of experimental paradigms, consistent with continuum, cognitive and evolutionary models of paranoia, that can guide decision-making in future research. Key clinical implications are the possibility of using experimental paradigms to investigate factors that ameliorate paranoid thinking in the context of threat or existing vulnerabilities.
